# Perspectives on the Probiotic Potential of Indigenous Moulds and Yeasts in Dry-Fermented Sausages

**DOI:** 10.3390/microorganisms11071746

**Published:** 2023-07-04

**Authors:** Micaela Álvarez, María J. Andrade, Eva Cebrián, Elia Roncero, Josué Delgado

**Affiliations:** Higiene y Seguridad Alimentaria, Instituto Universitario de Investigación de Carne y Productos Cárnicos, Facultad de Veterinaria, Universidad de Extremadura, Avda. de las Ciencias s/n, 10003 Cáceres, Spain; maalvarezr@unex.es (M.Á.); evcebrianc@unex.es (E.C.); eroncerob@unex.es (E.R.); jdperon@unex.es (J.D.)

**Keywords:** functional dry-fermented sausages, fungi, probiotics, postbiotics

## Abstract

The role of indigenous fungi in the appropriate development of sensory properties and the safety of dry-fermented sausages has been widely established. Nonetheless, their applications as probiotic agents have not been elucidated in such products yet, despite their promising functional features. Thus, it should be interesting to evaluate the probiotic potential of native *Debaryomyces hansenii* isolates from dry-fermented sausages and their application in the meat industry, because it is the most frequently isolated yeast species from these foodstuffs and its probiotic effects for animals as well as its possible probiotic activity for human beings have been demonstrated. Within the functional ability of foodborne yeasts, anti-inflammatory, antioxidant, antimicrobial, antigenotoxic, and immunomodulatory properties have been reported. Similarly, the use of dry-fermented sausages as vehicles for probiotic moulds remains a challenge because the survival and development of moulds in the gastrointestinal tract are still unknown. Nevertheless, some moulds have been isolated from faeces possibly from their spores as a form of resistance. Additionally, their beneficial effects on animals and humans, such as the decrease in lipid content and the anti-inflammatory activity, have been reported, although they seem to be more related to their postbiotic capacity due to the generated bioactive compounds with profunctional attributes than to their role as probiotics. Therefore, further studies providing knowledge useful for generating dry-fermented sausages with improved functionality are fully necessary.

## 1. Introduction 

Dry-fermented meat products are consumed and appreciated over the world and constitute a source of proteins and microelements [1]. Additionally, the environmental conditions at which these products are matured to allow the development of several microbial groups contribute to their transformation during the ripening process. These microbial groups are (a) Lactic Acid Bacteria (LAB), with powerful fermentation ability, decreasing the pH to achieve the desired texture, among other required sensory characteristics [2]; (b) Gram + Catalase + Cocci (GCC+), endowed with nitrate and nitrite reductase activities, as well as antioxidant properties by the mentioned catalase production, which contribute to colour formation and maintenance [3]; (c) yeasts, with proteolytic and lipolytic activities that generate sapid compounds, such as peptides, amino acids, and free fatty acids, which in turn are further transformed into new compounds with a positive impact on the sensory characteristics; and (d) moulds, also with proteolytic and lipolytic activities, as well as surrounding and covering the dry-fermented meat products provoking a slow maturation linked to a high sensory quality [4].

All these microorganisms have been described to be intentionally added to dry-fermented sausages as starter and/or protective cultures. Before being used, their safety must be checked. Those microorganisms considered under the qualified presumption of safety (QPS) by the European Food Safety Authority [5] or generally recognised as safe (GRAS) by the U.S. Food and Drug Administration [6] can be applied without additional tests. However, for those not included on this list, different safety analyses must be carried out beforehand [7].

Furthermore, once the microorganisms have the QPS/GRAS status or passed all the required safety tests, the previously mentioned four microbial groups could also be used as potential probiotic sources in dry-fermented sausages, because they are naturally adapted to develop in these matrices, reaching high levels [2,8,9]. Nevertheless, not only the high levels of microorganisms to be ingested, but also the survival in the gastrointestinal tract due to their resistance to acids and bile salts and adhesion to enterocytes are required to be considered appropriate probiotics [7,10]. Additionally, nutraceutical abilities and control of foodborne bacteria and fungi have to be evaluated in these microorganisms [11], with the ability to play a further positive role beyond the probiotic effects.

Among these microbial groups, LAB have been extensively evaluated for their probiotic potential [12,13]. In fact, there are multiple LAB strains described as probiotics in foodstuffs, and many of them have proven to be suitable for use in dry-fermented meat products. Nonetheless, the remaining three groups—GCC+, yeasts, and moulds—have been studied to a considerably lesser extent globally, particularly in the context of dry-fermented sausages. Scarce bibliography is thus found when linking them to probiotic activity in such matrices. Among them, only a GCC+ has been described as a probiotic in relation to dry-meat products [14]. However, to the best of our knowledge, the probiotic effects of autochthonous fungi from these products have not been checked yet.

Apart from the probiotic potential, the postbiotic effect can be understood as positive attributes rendered by microbial bioactive compounds, instead of the activity performed directly by the microorganisms in the body [11]. In contrast to probiotics, the microorganisms do not have to be alive or functional after their intake because the functional effect is exerted by the generated compounds in the foodstuffs. This has been mostly related to mould action [15,16,17], because their activity as probiotics can be considered negligible.

The long-term consumption of dry-fermented meat products has been associated with chronic diseases, such as colorectal cancer, blood vessel blockage, high blood pressure, and arteriosclerotic metabolite promotion [18,19]. The inclusion of probiotic microorganisms, other than the well-known LAB, in dry-fermented sausages could improve such an impact on consumer health [20] and, as a consequence, their acceptance. Thus, the use of fungi to achieve healthier meat products, endowed with functional properties by their postbiotic content or the use of these products as vehicles for the intake of fungi with probiotic activity, deserves to be studied.

Therefore, this review aims to collect current knowledge about the fungi native to dry-fermented sausages or compatible with them, provided with possible probiotic ability, as well as the challenges that remain to be performed to maximise their use in the meat industry.

## 2. Fungi in Dry-Fermented Sausages

The surface of dry-fermented sausages is naturally colonised by yeasts and moulds during their ripening. Some yeasts and moulds can provoke a wide variety of human diseases, such as allergic reactions and fungemia. However, these infections due to the consumption of food with these microorganisms are infrequent [21]. On the other hand, both their positive and negative effects on the sensory and safety attributes have been widely described and will be summarised below.

### 2.1. Yeasts

Dry-fermented sausages constitute an optimal substrate for the growth of yeasts due to their physicochemical characteristics, such as their pH as well as salt- and carbon-rich compound content [22], being *Debaryomyces hansenii* (teleomorph of *Candida famata*) the predominant species [22,23,24,25,26,27,28,29]. *Candida zeylanoides* and *Yarrowia lipolytica* have also been isolated to a lesser extent [22]. Apart from those previously mentioned, other genera have been isolated from different types of dry-fermented sausages, such as *Pichia*, *Rhodotorula*, *Cryptococcus*, *Saccharomyces,* and *Trichosporon* [24,25]. Normally, the amount of these naturally occurring yeasts in dry-fermented sausages is around 10^6^ cfu/g [25,30,31,32,33].

A positive effect on the organoleptic characteristics of dry-fermented sausages has been associated with some of their native yeasts [22,23,25,34]. For this reason, several studies have been focused on the intended inoculation of *D. hansenii*, promoting colour development through oxygen absorption and proteolytic activity (Table 1), which in turn results in the production of positive volatile compounds [23,35,36,37,38,39,40]. These volatile compounds are also the result of lipid oxidation and carbohydrate fermentation [35], producing compounds, such as 3-methylbutanol, 3-methylbutanal, and 2-propanone, which give dry-fermented sausages their highly appreciated characteristic aroma.

Another interesting technological yeast property with positive effects on the sensory characteristics of dry-fermented sausages is its lipolytic activity [25] and its ability to degrade peroxides [22,41] (Table 1).

Another important property of *D. hansenii* as a dominant species in dry-fermented sausages, is its antimicrobial capacity [24,36]. Currently, the use of protective cultures in the meat industry is mostly limited to the antifungal capacity of bacteria, such as LAB [43]. However, yeasts isolated from meat products are also being evaluated for their potential as inhibitors of toxigenic microorganisms with a possible application in the meat industry [44,45,46,47,48]. One of the main hazards to be addressed using *D. hansenii* as a protective culture is that posed by toxigenic moulds because this yeast has been shown to have an impact on the metabolism of *Penicillium verrucosum* [48,49], *Penicillium nordicum* [44,45,46,50], *Penicillium expansum* [36], *Aspergillus niger* [36], *Aspergillus parasiticus* [51,52], *Fusarium graminearium* and *Penicillium crustosum* [24], *Penicillium griseofulvum* [53], and *Aspergillus westerdijkiae* [54] (Table 2). Additionally, *D. hansenii* has displayed activity against some pathogenic yeasts, such as *Candida albicans* [36]. Different mechanisms of its antagonistic action have been established for *D. hansenii* (Table 2). In addition, *C. zeylanoides* has also been shown to have antifungal activity against *P. nordicum* and *A. westerdijkiae* in simulant media of fermented sausages [55] (Table 2).

### 2.2. Moulds

The moulds that most frequently colonise the surface of dry-fermented sausages belong to the genera *Penicillium* and *Aspergillus* [58,59,60]. Some of these moulds have shown a positive impact on their sensory characteristics (Table 3). The mycelial layer gives a characteristic external appearance to dry-fermented sausages, creating a microclimate by regulating moisture loss and protecting them from light and oxygen, thus reducing fat oxidation and subsequent rancidity [61]. This antioxidant effect is enhanced by the production of catalase [62]. On the other hand, moulds contribute to the proteolytic and lipolytic changes that occur during ripening [63], causing an increase in the concentration of amino acids and free fatty acids, which are precursors of desirable volatile compounds in dry-fermented sausages [10]. As a consequence, these activities contribute to the development of their characteristic flavour [64]. In this sense, *Penicillium aurantiogriseum* and *Penicillium camemberti* are some of the moulds that positively impact on the organoleptic characteristics in the final products (Table 3), both being used as starter cultures in sausage ripening [10]. Furthermore, *Penicillium chrysogenum* and *Penicillium nalgiovense* are responsible for the covering and ripening of sausages, resulting in a more consistent taste and flavour as well as in a more uniform appearance [10,65]. In addition, *P. aurantiogriseum*, despite not being native, has also been shown to improve the odour and flavour of dry-fermented sausages due to their increase in the production of desirable volatile compounds and other compounds derived from mould metabolism [64] (Table 3). Moreover, less lipid oxidation was also achieved due to its antioxidant activity [64].

Although *P. camemberti* coats the rind of some cheeses [66], it has also been proposed as a potential starter culture for dry-fermented sausage production [67]. Concretely, this mould provokes intense proteolysis and lipolysis, which results in an increase in the concentration of volatile compounds, free amino acids, and free fatty acids with subsequent flavour improvement (Table 3).

Furthermore, Hierro et al. [68] tested the effect of *Mucor racemosus*, *P. aurantiogriseum,* and *P. camemberti* isolated from Spanish fermented sausages and showed that their applications as starter cultures in dry-fermented sausages provoked a different flavour in the final product and protected against lipid oxidation (Table 3).

Despite the positive impact of mould populations on dry-fermented sausages, some of them can provoke unwanted effects, such as the alteration of their sensory characteristics. Thus, *Cladosporium oxysporum* has been described as being responsible for the formation of undesirable black spots on dry-fermented sausages [69]. In addition, some species belonging to the genus *Mucor* can produce a strong ammonia odour due to their high proteolytic activity [61]. Other undesirable moulds are those having the ability to produce secondary metabolites, such as mycotoxins [63,69]. In this sense, the most worrying mycotoxin in dry-fermented sausages is ochratoxin A (OTA) [70,71], with *P. nordicum* being the most frequent ochratoxigenic species isolated from them [60]. Additionally, *A. westerdijkiae* has also been described as a potential OTA producer in dry-fermented sausages [65].

On the other hand, autochthonous moulds can be used as protective cultures to control the growth of undesirable moulds. Therefore, certain strains of the former are capable of producing antifungal proteins against mycotoxin-producing moulds [52,72,73]. Specifically, *Penicillium allii-sativi* CECT 20922 (formerly *P. chrysogenum* CECT 20922) has been shown to produce the PgAFP protein, which confers to the mould the ability to reduce the growth of toxigenic moulds as well as OTA production [57] (Table 3). Apart from being used as a starter culture on the casings of dry-fermented sausages, *P. nalgiovense* may also be applied as a protective culture. Bernáldez et al. [8] thus reported the efficacy of *P. nalgiovense* to prevent the presence of toxigenic moulds and their mycotoxins in these kinds of products (Table 3).

**Table 3:** Main positive effects described for moulds in dry-fermented sausages.

Microorganism	Starter/Protective Culture	Activity	References
*Penicillium aurantiogriseum*	Starter	Increase in odour and flavour	[64]
*Penicillium camemberti*	Starter	Improvement in odour and flavour	[67]
*Mucor racemosus*	Starter	Ester formation Control of lipid oxidation	[68]
*P. aurantiogriseum*	Starter	Amino acid degradation Control of lipid oxidation	
*P. camemberti*	Starter		
*Penicillium chrysogenum*	Protective	Production of antifungal protein against toxigenic moulds	[52,72,73]
*Penicillium nalgiovense*	Protective	Reduction in ochratoxin A level	[8]

## 3. Probiotic Properties of Yeasts from Dry-Fermented Sausages

Although LAB are the main probiotic microorganisms selected by food industries as previously mentioned, yeasts have been reported as also having promising functional properties. Apart from improving the intestinal microbial balance, yeasts may synthesise metabolites in the fermented food with potential dietary and therapeutic importance. Within the functional abilities of yeasts, health-promoting and biotherapeutic properties (prevention and treatment of gastrointestinal infections, type II diabetes, and several cancers and adjusting intestinal microbiota and supporting healthy mucosal and immune system functions), nutraceutical abilities (cholesterol assimilation, degradation of unwanted/antinutrient compounds, and production of antioxidants or increase in their bioavailability), and improvement in food quality and safety (biological control of foodborne bacteria and fungi, degradation and removal of mycotoxins and bacterial toxins, food bio-fortification, and increase in the sensory scores) have been stated [11]. Furthermore, it is also noticeable that yeasts from food are rarely (if ever) associated with outbreaks or cases of foodborne illness [74].

Despite these features, only *Saccharomyces boulardii* and *Kluyveromyces fragilis* B0399 are currently used as probiotics for humans [11,75]. Perhaps the detrimental effects of these eukaryotic microorganisms on the shelf life and sensory properties due to their spoilage of many foodstuffs are responsible for their scarce use as probiotics despite their pivotal potential. Nonetheless, there is an increasing interest to characterize the probiotic properties of yeasts and their applications in foods [11].

Data about the probiotic potential of non-*Saccharomyces* and non-*Kluyveromyces* yeasts from fermented food are also available. Such yeasts include the species *D. hansenii* and *Y. lipolytica*, both found in dry-fermented sausages [22], with their high colonisation by the former, as mentioned in Section 2.1, being noteworthy. While the safety status of *D. hansenii* has been determined, *Y. lipolytica* can only be applied for production purposes when this implies the absence of its viable cells in the final product and food and feed products based on microbial biomass [5]. The usefulness of native *D. hansenii* strains as both starter and protective cultures has been reported for fermented meat products [23,46,56], but its probiotic effect has not been evaluated yet. However, the probiotic potential of *D. hansenii* isolated from cheeses has been confirmed, although it was strictly strain-dependent, which could lead to different effects [76]. Regarding its survival in gastrointestinal tract conditions, some of the strains were able to tolerate some of the *in vitro* stresses under microaerobic and/or anaerobic conditions and could have a potential to reach the gut alive (Table 4). Their adhesion to the surfaces of three models of intestinal epithelial surfaces (differentiated Caco-2 cells, undifferentiated Caco-2 cells, and mucin) was also checked with that of undifferentiated Caco-2 cells showing the best results. Even some strains adhered more strongly to the Caco-2 cells and mucin than the tested *S. boulardii* strains. In addition, some *D. hansenii* strains triggered a higher production of cytokine secretions in human dendritic cells than the established probiotic yeast *S. boulardii* [76]. Differences at the strain level were also established by Merchán et al. [77] when evaluating the functional properties of *D. hansenii* isolated from two Spanish soft paste cheeses (Table 4). The *D. hansenii* strain 414 isolated from feta cheese, which is able to resist the stress imposed by bile salts and to adhere to Caco-2 cells, has also been reported [78], suggesting its ability to colonise the intestine. However, such a dairy isolate did not display an appropriate capacity to survive at a low pH and modulate the immune responses, apart from exhibiting a low potential to reduce blood cholesterol and no antimicrobial activity. Interestingly, most of the *D. hansenii* isolates from Pecorino Abruzzese cheese had the potential ability to express antigenotoxic and antioxidative activities, together with acid–bile tolerance, being expected to reach the gut in a viable form and thus reducing the potential genotoxic risk due to mutagenic and genotoxic compounds [79] (Table 4). Furthermore, valuable functional traits were found in *D. hansenii* isolated from cheeses in the Italian Marche region [80] (Table 4). In addition to cheeses, the probiotic features of *D. hansenii* isolated from other fermented foodstuffs have been described (Table 4). Accordingly, all the *D. hansenii* strains (15) isolated from Greek-style black olives were able to grow at a low pH, and a vast majority of them tolerated the presence of bile salts (Table 4), which indicates their probiotic potential [81]. Additionally, some of them showed antimicrobial action against foodborne pathogens (Table 4). When Jeong et al. [82] evaluated the probiotic potential of two strains of *D. hansenii* isolated from Korean soy sauce, both displayed viability in the presence of bile salt and at a low pH and beneficial immunomodulatory activity, with the potential to suppress overactivated inflammatory responses. Additionally, they showed neither virulence nor acute oral toxicity (Table 4).

Although knowledge is scarce about the probiotic features of *D. hansenii* from food origins, its use as a feed additive for animals has shown beneficial health effects. Specifically, there is a broad range of data on *D. hansenii* as a probiotic in fish farming [83,84]. Some of the therapeutic traits exerted by *D. hansenii* include positive modulation of the immunomodulation, antioxidant activity, and growth enhancement of beneficial intestinal bacteria (Table 4).

Due to the production of compounds with functional properties, *D. hansenii* could also be used for fortifying foods with postbiotics. Therefore, *D. hansenii*-derived β-glucans modulate the innate immune response in goat leukocytes [85].

**Table 4:** Beneficial characteristics described for *Debaryomyces hansenii* as potential probiotic.

Source	Models	Properties	References
Cheese	Caco cells, human dendritic cells	Tolerance to gastrointestinal tract conditions Adhesion to Caco-2 cells and mucin Cytokine production (anti-inflammatory effect)	[76]
Spanish soft paste cheese	-	Tolerance to gastrointestinal tract conditions Autoaggregation ability Antioxidant activity	[77]
Feta cheese	Caco cells, mouse/rat spleen cells	Tolerance to bile salts Adhesion to Caco-2 cells No plasmid DNA	[78]
Pecorino Abruzzese cheese	*Escherichia coli* PQ37, HT-29 enterocytes	Tolerance to acid and bile Antioxidant activity Antigenotoxic activity	[79]
Greek-style black olives	-	Tolerance to low pH and bile salts at pH 8	[81]
Gut of rainbow trout	European sea bass larvae (*Dicentrarchus labrax*)	Antioxidant activity	[83]
Korean soybean fermented soy sauce “ganjang”	Human dendritic cells, mice	Tolerance to low pH and bile salts Immunomodulatory activity through induction of high levels of the anti-inflammatory cytokine IL-10 Nonvirulence without acute oral toxicity	[82]
Intestine of rainbow trout	Mice	Improvement in the immune innate response through enhanced phagocytic and respiratory burst activities, production of nitric oxide, IgA, and IgG; and upregulated gene expression of pro-inflammatory cytokines	[86]
Intestine of rainbow trout	Goat	Tolerance to acid pH Adhesion to goat intestine Antioxidant activity Improvement in the immune innate response through enhanced respiratory burst activity and production of nitric oxide Stimulation of the expression of immune-related gene signalling pathways	[87]
Lemon fruit	Gilthead seabream	Tolerance to acid pH Improvement in the immune innate response through enhanced respiratory burst activity and positive modulation of immune-related genes	[88]
Marine origin	Gilthead seabream	Tolerance to salt, acid pH, and bile salts Antimicrobial activity against pathogenic bacteria in aquaculture (*Aeromonas hydrophila*, *Vibrio vulnificus*, *Vibrio parahaemolyticus,* and *Photobacterium damselae*) Improvement in the immune response through effect on leucocyte phagocytic capacity, respiratory burst activity, peroxidase activity, serum C3 and IgM levels, and regulation of cytokine gene expression	[89]
Mouse intestine	Mice	Increase in the operational taxonomic units of intestinal bacteria Promotion of the growth of beneficial intestinal bacteria	[90,91]

## 4. Probiotic Properties of Moulds from Dry-Fermented Sausages

There are few studies about the probiotic capacity of foodborne moulds mainly due to the few species considered GRAS [6] and their lack of survival in the gastrointestinal tract, as they are aerobic microorganisms. Although some studies have detected *Penicillium* spp., *Aspergillus* spp., and *Cladosporium* spp. within the human gut microbiota [92,93], their presence in this tract cannot be assumed as the exertion of a given desired activity herein.

While there are no studies about the probiotic potential of moulds from dry-fermented sausages, some recent work in other fermented products indicate the beneficial effects of moulds, in some cases isolated from food matrices, such as rice and tea, in animals and humans (Table 5). Nonetheless, such positive properties linked to the consumption of foods manufactured with moulds seem to be related to their postbiotic ability instead of their probiotic one, because there are some bioactive compounds generated by these fungi that have demonstrated functional attributes [94]. Some of these compounds are antimicrobial substances as well as proteolytic and lipolytic enzymes that may help to digest the food, increasing the nutrient availability [94]. However, because to the best of our knowledge there are no studies focused on the postbiotic metabolites from moulds, the mechanisms involved in their beneficial activity are not exactly known.

Regarding the functional attributes of moulds, species belonging to the *Monascus* genus are frequently used in traditional Chinese medicine to ferment rice to obtain red mould rice, also known as red koji. In mice, the ingestion of monascin from red mould rice improved intestinal bacterial dysbiosis and repaired liver damage through the regulation of the transcription of genes involved in the lipid metabolism, antioxidative status, and inflammatory response, as well as the production of metabolites, such as taurine and riboflavin [15] (Table 5). Wistar rats fed with *Monascus*-fermented products decreased their glucose level due to the increase in insulin secretion, and thus these products are considered a useful tool for diabetes treatment [15] (Table 5). Similarly, *Aspergillus cristatus* isolated from Fuzhuan brick tea has been described as a potential probiotic due to its antibacterial properties against intestinal pathogens and its adhesion to human colon cells and gastrointestinal survival in mice [95] (Table 5). The mould *Chrysonilia crassa* decreased the serum low-density lipoprotein (LDL) concentration and alanine aminotransferase, enhancing the health of broilers supplemented with fermented rice by the mould [16] (Table 5). *Aspergillus awamori* added to the diet of broilers decreased the abdominal fat, plasma cholesterol, lipid peroxidation, and glutamic–oxaloacetic transaminase activity [96] (Table 5). *Aspergillus oryzae* has also been tested as a probiotic in poultry, although it is known that the mould cannot survive in anaerobic conditions, and consequently its activity is due to its postbiotic action [97]. This species is able to improve the digestibility and intestinal health in broilers and inhibits cholesterol biosynthesis in human hepatic T9A4 cells [94,98] (Table 5). The diet of Nile tilapia fishes supplemented with *A. oryzae* improved the defence against hypoxia stress by increasing the antioxidative enzymes superoxide dismutase and glutathione peroxidase and the immunomodulation by the decrease in glucose and cortisol [17] (Table 5). Fermented brown rice and rice brands with such mould species also showed anti-inflammatory and antimutagenic activities in mice [99] (Table 5). The fermented soybeans “tempeh” elaborated with *Rhizopus oligosporus* decreased the glycated haemoglobin A1 and the triglyceride concentrations of type II diabetic patients when the “tempeh” was encapsulated [100] (Table 5).

**Table 5:** Moulds with potential postbiotic activity in animals or humans.

Mould	Food	Model	Main Findings	References
*Monascus* spp.	Red koji	Mice	Improvement in intestinal dysbiosis Repair of liver damage Reduction in lipids Antioxidative activity Anti-inflammatory activity	[15]
*Monascus* spp.	Feed	Wistar rats	Decrease in glucose	[15]
*Aspergillus cristatus*	Fuuzhuan brick tea	Human colon cells and mice	Antimicrobial activity	[95]
*Chrysonilia crassa*	Rice	Broilers	Decrease in low-density lipoprotein Decrease in alanine aminotransferase	[16]
*Aspergillus awamori*	Feed	Broilers	Decrease in fat and cholesterol Decrease in lipid peroxidation Decrease in glutamic–oxaloacetic transaminase activity	[96]
*Aspergillus oryzae*	Feed	Broilers	Improvement in digestibility	[94]
*A. oryzae*	Feed	Broilers	Decrease in cholesterol	[98]
*A. oryzae*	Feed	Nile tilapia	Antioxidant activity Increase in immunomodulation	[17]
*A. oryzae*	Brown rice and rice brands	Mice	Anti-inflammatory activity Antimutagenic activity	[99]
*Rhizopus oligosporus*	“Tempeh”	Humans	Decrease in glycated hemoglobin Decrease in triglycerides	[100]

## 5. Future Perspectives and Conclusions

Because dry-fermented meat products have been linked to different diseases, strategies to improve their composition or transform them into functional foods deserve to be exploited. Fungi colonising these products can be an excellent tool for achieving this challenge.

Focusing on yeasts, *D. hansenii* is the pre-eminent one in dry-fermented meat products. Therefore, its probiotic potential deserves to be further explored to satisfy consumer demand and the competitiveness of this industry. It is worth noting that *D. hansenii*’s functional properties could vary depending on individual strains.

When referring to moulds, their probiotic effect is rather limited due to the anaerobic environment in the gastrointestinal tract. Then, their proved postbiotic effect could maximise the enhancement of these products. For this microbial group, two possible strategies could be followed. On the one hand, those moulds able to colonise dry-fermented sausages could be directly inoculated on their surface, and their postbiotic production should be evaluated. On the other hand, in those moulds whose growth and physiological requirements do not fit with the dry-fermented sausages, their inclusion after growing in a suitable matrix under the required conditions should be explored.

Both strategies based on fungi seek to resolve meat industry challenges and transform their products into healthier ones. Additionally, these tools can be considered within the clean label movement and are highly appreciated by consumers.

## Figures and Tables

**Table 1:** Effects of yeasts as starter cultures on dry-fermented sausages.

Microorganism	Main Activity	References
*Debaryomyces hansenii*	Flavour development by proteolysis	[39]
*D. hansenii*	Degradation of peroxides	[22,41]
*D. hansenii*	Production of volatile compounds	[23,35,36,37,38]
*Yarrowia lipolytica*	Lipolytic activity	[25]
*Debaryomyces* spp.	Volatile compound formation (inhibition of generation of compounds from lipid oxidation and promotion of ethyl esters formation)	[37]
*D. hansenii*	Production of meaty aromas (decrease in methional and methyl 2-methyl-3-furyl disulfide content and increase in methionol level)	[42]
*Debaryomyces* spp.	Lipolytic and proteolytic activities	[27]

**Table 2:** Effect of yeasts as protective cultures on dry-cured meat products.

Microorganism	Food Matrix	Target Microorganism	Main Activity	References
*Debaryomyces hansenii*	Dry-fermented sausages	*Penicillium verrucosum*	Competition for space and nutrients Production of metabolites	[48]
*D. hansenii*	Dry-fermented sausages	*Penicillium nordicum*	Damage to cell wall integrity	[56]
*D. hansenii*	Dry-cured meat environmental and nutritional conditions	*P. nordicum*	Production of extracellular compounds	[44]
*D. hansenii*	Dry-fermented sausages	*Aspergillus parasiticus*	Repression of genes involved in the aflatoxin biosynthetic pathway	[51]
*D. hansenii*	Dry-fermented sausages	*Fusarium graminearum* *A. parasiticus*	Production of metabolites	[24]
*D. hansenii*	Dry-fermented sausages medium	*Penicillium griseofulvum*	Competition for space	[52]
*D. hansenii*	Dry-fermented sausages	*P. nordicum*	Repression of the *otapksPN* and *otanpsPN* genes involved in the ochratoxin A (OTA) biosynthetic pathway	[57]
*Candida zeylanoides*	Dry-fermented sausages	*P. nordicum* *Aspergillus westerdijkiae*	Mould growth inhibition and OTA biosynthesis	[55]
*D. hansenii*	Dry-fermented sausages medium	*A. westerdijkiae*	Reduction in OTA production	[54]

## References

[1] Pereira P.M.d.C.C., Vicente A.F.d.R.B. (2013). Meat nutritional composition and nutritive role in the human diet. Meat Sci..

[2] Lizaso G., Chasco J., Beriain M.J. (1999). Microbiological and biochemical changes during ripening of salchichón, a Spanish dry cured sausage. Food Microbiol..

[3] Ordóñez J.A., Hierro E.M., Bruna J.M., De La Hoz L. (1999). Changes in the components of dry-fermented sausages during ripening. Crit. Rev. Food Sci. Nutr..

[4] Martín A., Córdoba J.J., Aranda E., Córdoba M.G., Asensio M.A. (2006). Contribution of a selected fungal population to the volatile compounds on dry-cured ham. Int. J. Food Microbiol..

[5] EFSA (European Food Safety Authority) European Food Safety Authority. https://www.efsa.europa.eu/en/topics/topic/qualified-presumption-safety-qps.

[6] Food and Drug Administration Microorganisms and Derived Ingredients Used in Food (Partial List). https://www.fda.gov/food/generally-recognized-safe-gras/microorganisms-microbial-derived-ingredients-used-food-partial-list.

[7] Li M., Wang Y., Cui H., Li Y., Sun Y., Qiu H.J. (2020). Characterization of lactic acid bacteria isolated from the gastrointestinal tract of a wild boar as potential probiotics. Front. Vet. Sci..

[8] Bernáldez V., Córdoba J.J., Rodríguez M., Cordero M., Polo L., Rodríguez A. (2013). Effect of *Penicillium nalgiovense* as protective culture in processing of dry-fermented sausage “salchichón”. Food Control.

[9] Cruxen C.E.d.S., Funck G.D., Haubert L., Dannenberg G., de Lima Marques J., Chaves F.C., da Silva W.P., Fiorentini Â.M. (2019). Selection of native bacterial starter culture in the production of fermented meat sausages: Application potential, safety aspects, and emerging technologies. Food Res. Int..

[10] Sunesen L.O., Stahnke L.H. (2003). Mould starter cultures for dry sausages—Selection, application and effects. Meat Sci..

[11] Sadeghi A., Ebrahimi M., Shahryari S., Kharazmi M.S., Jafari S.M. (2022). Food applications of probiotic yeasts; focusing on their techno-functional, postbiotic and protective capabilities. Trends Food Sci. Technol..

[12] Lahiri D., Nag M., Sarkar T., Ray R.R., Shariati M.A., Rebezov M., Bangar S.P., Lorenzo J.M., Domínguez R. (2022). Lactic Acid Bacteria (LAB): Autochthonous and probiotic microbes for meat preservation and fortification. Foods.

[13] Munekata P.E.S., Pateiro M., Zhang W., Domínguez R., Xing L., Fierro E.M., Lorenzo J.M. (2020). Autochthonous probiotics in meat products: Selection, identification, and their use as starter culture. Microorganisms.

[14] Borah D., Gogoi O., Adhikari C., Kakoti B.B. (2016). Isolation and characterization of the new indigenous *Staphylococcus* sp. DBOCP06 as a probiotic bacterium from traditionally fermented fish and meat products of Assam state. Egypt. J. Basic Appl. Sci..

[15] Wu L., Li W., Chen G., Yang Z., Lv X., Zheng L., Sun J., Ai L., Sun B., Ni L. (2022). Ameliorative effects of monascin from red mold rice on alcoholic liver injury and intestinal microbiota dysbiosis in mice. Food Biosci..

[16] Sugiharto S., Yudiarti T., Isroli I., Widiastuti E., Dwi Putra F. (2017). Effect of dietary supplementation with *Rhizopus oryzae* or *Chrysonilia crassa* on growth performance, blood profile, intestinal microbial population, and carcass traits in broilers exposed to heat stress. Arch. Anim. Breed..

[17] Dawood M.A.O., Eweedah N.M., Moustafa E.M., Farahat E.M. (2020). Probiotic effects of *Aspergillus oryzae* on the oxidative status, heat shock protein, and immune related gene expression of Nile tilapia (*Oreochromis niloticus*) under hypoxia challenge. Aquaculture.

[18] Cantwell M., Elliot C. (2017). Nitrates, nitrites and nitrosamines from processed meat intake and colorectal cancer risk. J. Clin. Nutr. Diet..

[19] Delgado J., Ansorena D., Van Hecke T., Astiasarán I., De Smet S., Estévez M. (2021). Meat lipids, NaCl and carnitine: Do they unveil the conundrum of the association between red and processed meat intake and cardiovascular diseases?. Meat Sci..

[20] Sirini N., Frizzo L.S., Aleu G., Soto L.P., Rosmini M.R. (2021). Use of probiotic microorganisms in the formulation of healthy meat products. Curr. Opin. Food Sci..

[21] Delgado J., Álvarez M., Cebrián E., Martín I., Roncero E., Rodríguez M. (2023). Biocontrol of pathogen microorganisms in ripened foods of animal origin. Microorganisms.

[22] Encinas J.P., López-Díaz T.M., García-López M.L., Otero A., Moreno B. (2000). Yeast populations on Spanish fermented sausages. Meat Sci..

[23] Andrade M.J., Córdoba J.J., Casado E.M., Córdoba M.G., Rodríguez M. (2010). Effect of selected strains of *Debaryomyces hansenii* on the volatile compound production of dry fermented sausage “salchichón”. Meat Sci..

[24] García-Béjar B., Sánchez-Carabias D., Alarcon M., Arévalo-Villena M., Briones A. (2020). Autochthonous yeast from pork and game meat fermented sausages for application in meat protection and aroma developing. Animals.

[25] Gardini F., Suzzi G., Lombardi A., Galgano F., Crudele M.A., Andrighetto C., Schirone M., Tofalo R. (2001). A survey of yeasts in traditional sausages of southern Italy. FEMS Yeast Res..

[26] Jiang L., Mu Y., Su W., Tian H., Zhao M., Su G., Zhao C. (2023). Effects of *Pediococcus acidilactici* and *Rhizopus oryzae* on microbiota and metabolomic profiling in fermented dry-cure mutton sausages. Food Chem..

[27] Mendonça R.C.S., Gouvêa D.M., Hungaro H.M., Sodré A.d.F., Querol-Simon A. (2013). Dynamics of the yeast flora in artisanal country style and industrial dry cured sausage (yeast in fermented sausage). Food Control.

[28] Mi R., Chen X., Xiong S., Qi B., Li J., Qiao X., Chen W., Qu C., Wang S. (2021). Predominant yeasts in Chinese Dong fermented pork (Nanx Wudl) and their aroma-producing properties in fermented sausage condition. Food Sci. Hum. Wellness.

[29] Osei Abunyewa A.A., Laing E., Hugo A., Viljoen B.C. (2000). The population change of yeasts in commercial salami. Food Microbiol..

[30] Cocolin L., Urso R., Rantsiou K., Cantoni C., Comi G. (2006). Dynamics and characterization of yeasts during natural fermentation of Italian sausages. FEMS Yeast Res..

[31] Perea-Sanz L., López-Díez J.J., Belloch C., Flores M. (2020). Counteracting the effect of reducing nitrate/nitrite levels on dry fermented sausage aroma by *Debaryomyces hansenii* inoculation. Meat Sci..

[32] Aquilanti L., Santarelli S., Silvestri G., Osimani A., Petruzzelli A., Clementi F. (2007). The microbial ecology of a typical Italian salami during its natural fermentation. Int. J. Food Microbiol..

[33] Di Cagno R., Chaves Lòpez C., Tofalo R., Gallo G., De Angelis M., Paparella A., Hammes W.P., Gobbetti M. (2008). Comparison of the compositional, microbiological, biochemical and volatile profile characteristics of three Italian PDO fermented sausages. Meat Sci..

[34] Corral S., Belloch C., López-Díez J.J., Salvador A., Flores M. (2017). Yeast inoculation as a strategy to improve the physico-chemical and sensory properties of reduced salt fermented sausages produced with entire male fat. Meat Sci..

[35] Bolumar T., Sanz Y., Flores M., Aristoy M.C., Toldrá F., Flores J. (2006). Sensory improvement of dry-fermented sausages by the addition of cell-free extracts from *Debaryomyces hansenii* and *Lactobacillus sakei*. Meat Sci..

[36] Chacón-Navarrete H., Ruiz-Pérez F., Ruiz-Castilla F.J., Ramos J. (2022). Exploring biocontrol of unwanted fungi by autochthonous *Debaryomyces hansenii* strains isolated from dry meat products. J. Fungi.

[37] Flores M., Durá M.A., Marco A., Toldrá F. (2004). Effect of *Debaryomyces* spp. on aroma formation and sensory quality of dry-fermented sausages. Meat Sci..

[38] Li L., Belloch C., Flores M. (2023). A comparative study of savory and toasted aromas in dry cured loins versus dry fermented sausages. LWT.

[39] Patrignani F., Iucci L., Vallicelli M., Guerzoni M.E., Gardini F., Lanciotti R. (2007). Role of surface-inoculated *Debaryomyces hansenii* and *Yarrowia lipolytica* strains in dried fermented sausage manufacture. Part 1: Evaluation of their effects on microbial evolution, lipolytic and proteolytic patterns. Meat Sci..

[40] Wen R., Yin X., Hu Y., Chen Q., Kong B. (2022). Technological properties and flavour formation potential of yeast strains isolated from traditional dry fermented sausages in Northeast China. LWT.

[41] Murgia M.A., Marongiu A., Aponte M., Blaiotta G., Deiana P., Mangia N.P. (2019). Impact of a selected *Debaryomyces hansenii* strain’s inoculation on the quality of Sardinian fermented sausages. Food Res. Int..

[42] Flores M., Perea-Sanz L., López-Díez J.J., Belloch C. (2021). Meaty aroma notes from free amino acids and thiamine in nitrite-reduced, dry-fermented, yeast-inoculated sausages. Food Chem..

[43] Nazareth T.d.M., Calpe J., Luz C., Mañes J., Meca G. (2023). Manufacture of a potential antifungal ingredient using lactic acid bacteria from dry-cured sausages. Foods.

[44] Álvarez M., Núñez F., Delgado J., Andrade M.J., Rodríguez M., Rodríguez A. (2021). Competitiveness of three biocontrol candidates against ochratoxigenic *Penicillium nordicum* under dry-cured meat environmental and nutritional conditions. Fungal Biol..

[45] Andrade M.J., Thorsen L., Rodríguez A., Córdoba J.J., Jespersen L. (2014). Inhibition of ochratoxigenic moulds by *Debaryomyces hansenii* strains for biopreservation of dry-cured meat products. Int. J. Food Microbiol..

[46] Cebrián E., Núñez F., Álvarez M., Roncero E., Rodríguez M. (2022). Biocontrol of ochratoxigenic *Penicillium nordicum* in dry-cured fermented sausages by *Debaryomyces hansenii* and *Staphylococcus xylosus*. Int. J. Food Microbiol..

[47] Delgado J., Peromingo B., Núñez F., Asensio M.A. (2016). Use of molds and their antifungal proteins for biocontrol of toxigenic molds on dry-ripened cheese and meats. Curr. Opin. Food Sci..

[48] Núñez F., Lara M.S., Peromingo B., Delgado J., Sánchez-Montero L., Andrade M.J. (2015). Selection and evaluation of *Debaryomyces hansenii* isolates as potential bioprotective agents against toxigenic penicillia in dry-fermented sausages. Food Microbiol..

[49] Peromingo B., Núñez F., Rodríguez A., Alía A., Andrade M.J. (2018). Potential of yeasts isolated from dry-cured ham to control ochratoxin A production in meat models. Int. J. Food Microbiol..

[50] Álvarez M., Andrade M.J., García C., Rondán J.J., Núñez F. (2020). Effects of preservative agents on quality attributes of dry-cured fermented sausages. Foods.

[51] Peromingo B., Andrade M.J., Delgado J., Sánchez-Montero L., Núñez F. (2019). Biocontrol of aflatoxigenic *Aspergillus parasiticus* by native *Debaryomyces hansenii* in dry-cured meat products. Food Microbiol..

[52] Delgado J., Rodríguez A., García A., Núñez F., Asensio M. (2018). Inhibitory effect of PgAFP and protective cultures on *Aspergillus parasiticus* growth and aflatoxins production on dry-fermented sausage and cheese. Microorganisms.

[53] Delgado J., Peromingo B., Rodríguez A. (2019). Biocontrol of *Penicillium griseofulvum* to reduce cyclopiazonic acid contamination in dry-fermented sausages. Int. J. Food Microbiol..

[54] Álvarez M., Núñez F., Delgado J., Andrade M.J., Rodrigues P. (2022). Proteomic evaluation of the effect of antifungal agents on *Aspergillus westerdijkiae* ochratoxin A production in a dry-cured fermented sausage-based medium. Int. J. Food Microbiol..

[55] Meftah S., Abid S., Dias T., Rodrigues P. (2018). Effect of dry-sausage starter culture and endogenous yeasts on *Aspergillus westerdijkiae* and *Penicillium nordicum* growth and OTA production. LWT-Food Sci. Technol..

[56] Álvarez M., Delgado J., Núñez F., Roncero E., Andrade M.J. (2022). Proteomic approach to unveil the ochratoxin A repression by *Debaryomyces hansenii* and rosemary on *Penicillium nordicum* during dry-cured fermented sausages ripening. Food Control.

[57] Cebrián E., Rodríguez M., Peromingo B., Bermúdez E., Núñez F. (2019). Efficacy of the combined protective cultures of *Penicillium chrysogenum* and *Debaryomyces hansenii* for the control of ochratoxin A hazard in dry-cured ham. Toxins.

[58] Pleadin J., Zadravec M., Brnić D., Perković I., Škrivanko M., Kovačević D. (2017). Moulds and mycotoxins detected in the regional speciality fermented sausage “slavonski kulen” during a 1-year production period. Food Addit. Contam. Part A.

[59] López-Díaz T.-M., Santos J.-A.A., García-López M.-L., Otero A. (2001). Surface mycoflora of a Spanish fermented meat sausage and toxigenicity of *Penicillium* isolates. Int. J. Food Microbiol..

[60] Iacumin L., Chiesa L., Boscolo D., Manzano M., Cantoni C., Orlic S., Comi G. (2009). Moulds and ochratoxin A on surfaces of artisanal and industrial dry sausages. Food Microbiol..

[61] Toldrá F., Astiasarán I., Sebranek J., Talon R. (2015). Handbook of Fermented Meat and Poultry.

[62] Leroy F., De Vuyst L., Caballero B., Finglas P.M., Toldrá F. (2015). Fermented Foods: Fermented Meat Products. Encyclopedia of Food and Health.

[63] Comi G., Iacumin L. (2013). Ecology of moulds during the pre-ripening and ripening of San Daniele dry cured ham. Food Res. Int..

[64] Bruna J.M., Hierro E.M., de la Hoz L., Mottram D.S., Fernández M., Ordóñez J.A. (2001). The contribution of *Penicillium aurantiogriseum* to the volatile composition and sensory quality of dry fermented sausages. Meat Sci..

[65] Iacumin L., Milesi S., Pirani S., Comi G., Chiesa L.M. (2011). Ochratoxigenic mold and ochratoxin A in fermented sausages from different areas in northern Italy: Occurrence, reduction or prevention with ozonated air. J. Food Saf..

[66] Kalai S., Anzala L., Bensoussan M., Dantigny P. (2017). Modelling the effect of temperature, pH, water activity, and organic acids on the germination time of *Penicillium camemberti* and *Penicillium roqueforti* conidia. Int. J. Food Microbiol..

[67] Bruna J.M., Hierro E.M., De La Hoz L., Mottram D.S., Fernández M., Ordóñez J.A. (2003). Changes in selected biochemical and sensory parameters as affected by the superficial inoculation of *Penicillium camemberti* on dry fermented sausages. Int. J. Food Microbiol..

[68] Hierro E., Ordóñez J.A., Bruna J.M., Pin C., Fernández M., De La Hoz L. (2005). Volatile compound generation in dry fermented sausages by the surface inoculation of selected mould species. Eur. Food Res. Technol..

[69] Lozano-Ojalvo D., Rodríguez A., Cordero M., Bernáldez V., Reyes-Prieto M., Córdoba J.J. (2015). Characterisation and detection of spoilage mould responsible for black spot in dry-cured fermented sausages. Meat Sci..

[70] Altafini A., Fedrizzi G., Roncada P. (2019). Occurrence of ochratoxin A in typical salami produced in different regions of Italy. Mycotoxin Res..

[71] Merla C., Andreoli G., Garino C., Vicari N., Tosi G., Guglielminetti M.L., Moretti A., Biancardi A., Arlorio M., Fabbi M. (2018). Monitoring of ochratoxin A and ochratoxin-producing fungi in traditional salami manufactured in Northern Italy. Mycotoxin Res..

[72] Acosta R., Rodríguez-Martín A., Martín A., Núñez F., Asensio M.A. (2009). Selection of antifungal protein-producing molds from dry-cured meat products. Int. J. Food Microbiol..

[73] Delgado J., Owens R., Doyle S., Asensio M.A., Núñez F. (2015). Impact of the antifungal protein PgAFP from *Penicillium chrysogenum* on the protein profile in *Aspergillus flavus*. Appl. Microbiol. Biotechnol..

[74] Querol A., Fleet G.H., Amparo Querol G.F. (2006). Yeasts in Food and Beverages.

[75] Menezes A.G.T., Ramos C.L., Cenzi G., Melo D.S., Dias D.R., Schwan R.F. (2020). Probiotic potential, antioxidant activity, and phytase production of indigenous yeasts isolated from indigenous fermented foods. Probiot. Antimicrob. Proteins.

[76] Ochangco H.S., Gamero A., Smith I.M., Christensen J.E., Jespersen L., Arneborg N. (2016). *In vitro* investigation of *Debaryomyces hansenii* strains for potential probiotic properties. World J. Microbiol. Biotechnol..

[77] Merchán A.V., Benito M.J., Galván A.I., Ruiz-Moyano Seco de Herrera S. (2020). Identification and selection of yeast with functional properties for future application in soft paste cheese. LWT.

[78] Kourelis A., Kotzamanidis C., Litopoulou-Tzanetaki E., Scouras Z.G., Tzanetakis N., Yiangou M. (2010). Preliminary probiotic selection of dairy and human yeast strains. J. Biol. Res..

[79] Trotta F., Caldini G., Dominici L., Federici E., Tofalo R., Schirone M., Corsetti A., Suzzi G., Cenci G. (2012). Food borne yeasts as DNA-bioprotective agents against model genotoxins. Int. J. Food Microbiol..

[80] Agarbati A., Marini E., Galli E., Canonico L., Ciani M., Comitini F. (2021). Characterization of wild yeasts isolated from artisan dairies in the Marche region, Italy, for selection of promising functional starters. LWT.

[81] Psani M., Kotzekidou P. (2006). Technological characteristics of yeast strains and their potential as starter adjuncts in Greek-style black olive fermentation. World J. Microbiol. Biotechnol..

[82] Jeong D.M., Yoo S.J., Jeon M.S., Chun B.H., Han D.M., Jeon C.O., Eyun S.I., Seo Y.J., Kang H.A. (2022). Genomic features, aroma profiles, and probiotic potential of the *Debaryomyces hansenii* species complex strains isolated from Korean soybean fermented food. Food Microbiol..

[83] Tovar-Ramírez D., Mazurais D., Gatesoupe J.F., Quazuguel P., Cahu C.L., Zambonino-Infante J.L. (2010). Dietary probiotic live yeast modulates antioxidant enzyme activities and gene expression of sea bass (*Dicentrarchus labrax*) larvae. Aquaculture.

[84] Angulo M., Reyes-Becerril M., Medina-Córdova N., Tovar-Ramírez D., Angulo C. (2020). Probiotic and nutritional effects of *Debaryomyces hansenii* on animals. Appl. Microbiol. Biotechnol..

[85] Angulo M., Reyes-Becerril M., Tovar-Ramírez D., Ascencio F., Angulo C. (2018). *Debaryomyces hansenii* CBS 8339 β-glucan enhances immune responses and down-stream gene signaling pathways in goat peripheral blood leukocytes. Dev. Comp. Immunol..

[86] Angulo M., Ramos A., Reyes-Becerril M., Guerra K., Monreal-Escalante E., Angulo C. (2023). Probiotic *Debaryomyces hansenii* CBS 8339 yeast enhanced immune responses in mice. 3 Biotech.

[87] Angulo M., Reyes-Becerril M., Cepeda-Palacios R., Tovar-Ramírez D., Esteban M.Á., Angulo C. (2019). Probiotic effects of marine *Debaryomyces hansenii* CBS 8339 on innate immune and antioxidant parameters in newborn goats. Appl. Microbiol. Biotechnol..

[88] Reyes-Becerril M., Ascencio-Valle F., Meseguer J., Tapia-Paniagua S.T., Moriñigo M.A., Esteban M.Á. (2012). *Debaryomyces hansenii* L2-enriched diet enhances the immunity status, gene expression and intestine functionality in gilthead seabream (*Sparus aurata* L.). Aquac. Res..

[89] Reyes-Becerril M., Angulo C., Angulo M., Esteban M.Á. (2021). Probiotic properties of *Debaryomyces hansenii* BCS004 and their immunostimulatory effect in supplemented diets for gilthead seabream (*Sparus aurata*). Aquac. Res..

[90] Wu Y., Tang Y., Xiao N.Q., Wang C.H., Tan Z.J. (2020). Bacterial lactase gene characteristics in intestinal contents of antibiotic-associated diarrhea mice treated with *Debaryomyces hansenii*. Med. Sci. Monit..

[91] He L., Long C., Liu Y., Guo Y., Xiao N., Tan Z. (2017). Effects of *Debaryomyces hansenii* treatment on intestinal microorganisms in mice with antibiotics-induced diarrhea. 3 Biotech.

[92] Hallen-Adams H.E., Kachman S.D., Kim J., Legge R.M., Martínez I. (2015). Fungi inhabiting the healthy human gastrointestinal tract: A diverse and dynamic community. Fungal Ecol..

[93] Hallen-Adams H.E., Suhr M.J. (2017). Fungi in the healthy human gastrointestinal tract. Virulence.

[94] Sugiharto S. (2019). A review of filamentous fungi in broiler production. Ann. Agric. Sci..

[95] Wang X., Cui Y., Sang C., Wang B., Yuan Y., Liu L., Yuan Y., Yue T. (2022). Fungi with potential probiotic properties isolated from Fuzhuan brick tea. Food Sci. Hum. Wellness.

[96] Saleh A.A. *Aspergillus awamori* as probiotic in broiler chickens. Proceedings of the 9th Asia Pacific Poultry Conference.

[97] KyungWoo L., Kee S., Lee B.D. (2006). *Aspergillus oryzae* as probiotic in poultry—A review. Int. J. Poult. Sci..

[98] Hajjaj H., Duboc P., Fay L.B., Zbinden I., Macé K., Niederberger P. (2005). *Aspergillus oryzae* produces compounds inhibiting cholesterol biosynthesis downstream of dihydrolanosterol. FEMS Microbiol. Lett..

[99] Nemoto H., Otake M., Matsumoto T., Izutsu R., Jehung J.P., Goto K., Osaki M., Mayama M., Shikanai M., Kobayashi H. (2022). Prevention of tumor progression in inflammation-related carcinogenesis by anti-inflammatory and anti-mutagenic effects brought about by ingesting fermented brown rice and rice bran with *Aspergillus oryzae* (FBRA). J. Funct. Foods.

[100] Su H.K., Tsai M.H., Chao H.R., Wu M.L., Lu J.H. (2021). Data on effect of Tempeh fermentation on patients with type II diabetes. Data Br..

